# Seasonal Restructuring of Microbial Communities and Resistomes in the Shitalakshya River, Bangladesh Revealed by Shotgun Metagenomics

**DOI:** 10.1002/mbo3.70359

**Published:** 2026-07-01

**Authors:** Muhammad Ehteshamul Haque, Md. Shaminur Rahman, Munawar Sultana, Anowara Begum

**Affiliations:** ^1^ Department of Microbiology University of Dhaka Dhaka Bangladesh; ^2^ Department of Microbiology Jashore University of Science and Technology Jashore Bangladesh

**Keywords:** antimicrobial resistance, co‐selection, microbial community dynamics, resistome, Shitalakshya River, shotgun metagenomics

## Abstract

Urban rivers supplying drinking water face mounting pollution and AMR threats. We combined shotgun metagenomics with physicochemical analysis to investigate microbial community and resistome dynamics in Bangladesh's Shitalakshya River, a drinking water source under increasing pollution pressure, during early and peak dry seasons. Peak dry season water quality deteriorated markedly, characterized by hypoxia and elevated nutrient and organic carbon levels, which drove pronounced restructuring of the river microbiome. A distinct shift occurred from *Myroides* dominance toward a more diverse assemblage enriched in pollution‐tolerant and opportunistic genera, notably *Comamonas, Brevundimonas, Tissierella*, and *Aeromonas*. Metagenomic profiling revealed a diverse resistome encompassing antibiotic, metal, and biocide resistance genes. Although overall antibiotic resistance gene abundance declined slightly, metal resistance genes increased more than twofold, with strong enrichment of mercury resistance determinants such as *merA*. Concurrent increases in multidrug efflux pump genes suggested potential co‐selection driven by metal and chemical stressors. These findings indicate that dry‐season pollutant concentration reshapes both microbial communities and resistance profiles through non‐antibiotic selective pressures. Despite limited sampling, this study provides a baseline metagenomic snapshot of antimicrobial resistance dynamics in a climate‐stressed urban river system, offering vital insights for pollution abatement and the safeguarding of drinking water safety.

## Graphical abstract

Dry‐season pollutant concentration drives microbial restructuring in Bangladesh's Shitalakshya River, shifting communities from *Myroides* to stress‐tolerant genera while enriching metal resistance genes and efflux pumps, revealing non‐antibiotic co‐selection as a key driver of antimicrobial resistance in urban waters.

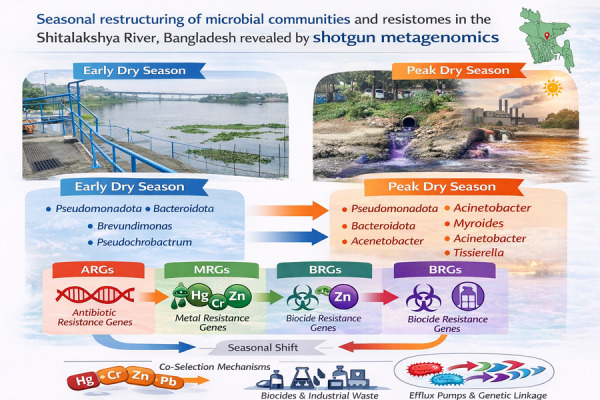

## 1 Introduction

Bangladesh, historically a riverine country, depends heavily on its waterways for socio‐economic stability. The Shitalakshya River serves as a vital lifeline for Dhaka, supplying raw water to the country's largest surface water treatment plant (Serajuddin et al. 2019a). Despite its strategic importance, the river has undergone severe environmental degradation over the past three decades due to untreated industrial effluents, municipal sewage, and agricultural runoff (Islam et al. 2025; Jolly et al. 2023; Majed and Islam 2022; Whitehead et al. 2019). These inputs create strong selective pressures that may influence microbial community composition and resistance gene dynamics in water directly linked to drinking‐water abstraction.

Microorganisms drive fundamental ecosystem functions in rivers, including nutrient cycling, organic matter mineralization, and contaminant transformation (Liu et al. 2012). Consequently, shifts in microbial community structure serve as sensitive indicators of ecosystem health and functional resilience (Galand et al. 2016; Santillan et al. 2019). Rivers in densely populated regions are increasingly recognized as critical reservoirs and conduits for antibiotic resistance genes (ARGs), now classified as emerging environmental pollutants by the World Health Organization (Reddy and Dubey 2019; Taylor et al. 2011; World Health Organization 2015). In Bangladesh, high population density, extensive antibiotic usage, inadequate wastewater treatment, and industrial metal contamination converge to promote the persistence and spread of antimicrobial resistance in surface waters (Sharif et al. 2025). The co‐occurrence of ARGs with metal resistance genes (MRGs) on mobile genetic elements (MGEs) facilitates co‐resistance, whereby non‐antibiotic stressors such as heavy metals maintain multidrug resistance within microbial communities (Chukwu et al. 2023; Engin et al. 2023; Gillieatt and Coleman 2024). Given that metals from tanneries, textile dyeing, and other industries are prevalent in Bangladeshi rivers (Jolly 2023), understanding co‐selection dynamics is essential for assessing AMR risks in drinking water sources.

Previous studies across South Asian rivers consistently report dominance of pollution‐tolerant bacterial groups, particularly *Pseudomonadota* (Proteobacteria), including genera such as *Pseudomonas*, *Aeromonas*, and *Acinetobacter*, which are well adapted to organic pollution, hypoxia, and toxic metal exposure (Adhikari et al. 2025; Akhtar and Malik 2025; Premke et al. 2020; Rout et al. 2022, 2023, 2024b). Bacteriophages also play a critical regulatory role in these environments, with diverse phage communities contributing to the control of pathogenic bacterial populations (Behera et al. 2023). However, comprehensive documentation of taxonomic composition across multiple ranks is essential to deciphering how a river's microbiome responds to varying environmental pressures (Knight et al. 2018). Less disturbed river sites tend to support more phylogenetically balanced communities with reduced abundances of stress‐resistant lineages (Lenart‐Boroń et al. 2022). Recent studies further demonstrate that environmental factors, including turbidity, nutrients, and metals, strongly influence microbial community assembly and ARG dissemination in aquatic systems (Faruk et al. 2025; Zhao et al. 2025; Mourão et al. 2025).

Despite growing evidence of AMR in Indian rivers (Rout et al. 2023; Behera et al. 2020a, 2020b), comparable metagenomic data from other South Asian countries, such as Bangladesh, remain sparse. Asaduzzaman et al. (2022) provided culture‐based and molecular evidence of AMR spatiotemporal distribution in Bangladeshi waters, yet comprehensive shotgun metagenomic analyses are still lacking. Shotgun metagenomics overcomes limitations of traditional culture‐based methods by enabling culture‐independent profiling of microbial taxa and resistance genes directly from environmental DNA (Chen et al. 2022; Rout et al. 2022, 2025). Metagenomic approaches have been applied to major South Asian rivers, including the Ganges and Brahmaputra, revealing strong linkages between pollution, microbial community restructuring, and resistance dynamics (Adhikari et al. 2025; Behera et al. 2020a, 2020b; Das et al. 2020; Rout et al. 2022, 2023, 2024a, 2024b, 2025). Despite its central importance to the Bangladesh drinking water supply, the Shitalakshya River remains largely unexplored using these tools, leaving critical knowledge gaps regarding microbial and resistance processes in this highly stressed urban river.

This study advances beyond previous South Asian River microbiome research by providing the first integrated metagenomic and hydrochemical evidence demonstrating that dry‐season pollutant concentration, rather than antibiotic input alone, is the dominant driver reshaping both taxonomic composition and multidrug resistance potential in a tropical urban river. Unlike broad seasonal comparisons reported for the Ganges and Brahmaputra (Adhikari et al. 2025; Das et al. 2020), this work isolates the effect of intensified dry‐season conditions within a single season, revealing rapid resistome restructuring mechanisms that have not been previously documented. By establishing baseline metagenomic snapshot under contrasting hydrological extremes, this work provides a methodological and conceptual framework for future longitudinal monitoring of climate‐stressed aquatic systems.

### 1.1 Hypothesis and Study Aims

We hypothesized that dry‐season reductions in river flow and concomitant increases in pollutant concentration would drive significant shifts in microbial community structure and enrich for stress‐tolerance and resistance determinants. To test this, we conducted an integrated physicochemical and metagenomic analysis at a single, highly impacted site during two hydrologically distinct periods: early dry season (December 2024) and peak dry season (February 2025). While sampling was limited to two time points, these periods represent starkly different flow and pollution regimes, offering a valuable high‐contrast snapshot of seasonal extremes. Specifically, this study aimed to: (a) characterize bacterial community composition at multiple taxonomic ranks, (b) quantify the abundance and diversity of ARGs, metal/biocide resistance genes, and (c) examine links between environmental parameters and microbiome/resistome profiles. Beyond ecological insight, this work addresses urgent water security challenges in Bangladesh. As climate change intensifies seasonal weather patterns, dry‐period low flows may further concentrate pollutants, exacerbating AMR risks. By establishing a baseline metagenomic profile, this study informs monitoring frameworks, supports pollution mitigation strategies, and contributes to a One Health approach for safeguarding river ecosystems and public health.

## 2 Materials and Methods

### 2.1 Study Area

The Shitalakshya River at the Sarulia intake point (23°43′02″ N, 90°30′00″ E), Narayanganj, Bangladesh, was selected as the representative study site (Supplementary Figure S1). This site functions as the primary raw water intake for the Saidabad Water Treatment Plant (Phase I and II), the country's largest surface water treatment facility, supplying drinking water to millions of residents in Dhaka. This midstream location lies downstream of major industrial and municipal discharge zones, including textile, dyeing, pharmaceutical, chemical, and food‐processing industries. Continuous effluent inputs contribute elevated organic matter, nutrients, metals, and xenobiotics, generating strong selective pressures that may influence microbial community composition, functional traits, and resistance gene dynamics.

### 2.2 Sample Collection and Rationale

Water sampling was conducted during two hydrologically distinct dry‐season periods, early dry season (December 2024) and peak dry season (February 2025), to capture contrasting environmental conditions associated with progressive flow reduction and pollutant concentration in the river system. Rather than representing continuous seasonal monitoring, these two targeted time points were selected to reflect hydrological extremes within the dry season, when reduced river discharge and diminished dilution capacity intensify anthropogenic pollution stress and alter microbial community dynamics. The environmental relevance of this sampling window is substantiated by operational evidence: during this period, deterioration of raw water quality at the intake of the Saidabad Water Treatment Plant (Phase I and II) necessitated the implementation of additional biological pre‐treatment prior to conventional treatment processes (Haque et al. 2025).

At each time point, surface water samples (upper 0.5 m) were collected from the midstream region at the drinking water abstraction zone using sterile 2 L high‐density polyethylene (HDPE) bottles. Triplicate water samples (*n* = 3 per site, 2 L each; 6 L total per site) were collected independently on the same day to account for local spatial and analytical variability within the sampling site. The triplicate design provides true field replicates that capture the inherent spatial micro‐heterogeneity of microbial communities at the sampling point. The midstream location was selected to minimize bank‐associated heterogeneity and to better represent the mixed water column entering the treatment plant intake. Samples were immediately placed on ice, transported to the laboratory, and stored at 4°C before processing to minimize biological alteration.

Each 2 L replicate was partitioned as follows: 1 L for DNA extraction, 500 mL for filtration (0.45 µm), and 500 mL unfiltered for physicochemical and TOC measurement. The 500 mL filtrate was further subdivided, with 200 mL acidified (pH < 2, HNO_3_) for dissolved metal analysis and the remaining 300 mL used for nutrient determination and UV_254_ absorbance measurement. This allocation ensured sufficient volume for all downstream analyses while maintaining true field replication.

Although sediment can serve as a long‐term reservoir and sink for pollutants, antibiotic resistance genes, and microbial contaminants in flowing river systems, the present study specifically focused on the water column because it directly represents the fraction abstracted for drinking water treatment and therefore poses the most immediate public health relevance.

The objective of this work was to characterize short‐term microbial and resistome responses in intake water under acute dry‐season stress conditions rather than long‐term contaminant accumulation in benthic environments. Nevertheless, sediment‐associated resistome dynamics remain important and should be incorporated into future longitudinal investigations to provide a more comprehensive understanding of pollutant persistence and environmental reservoirs of antimicrobial resistance in the river ecosystem.

This targeted paired‐sampling design provides a high‐contrast snapshot of dry‐season environmental stress, enabling the identification of key microbial community and resistome responses under acute pollution conditions while maintaining direct operational relevance to drinking water safety monitoring at the point of abstraction.

### 2.3 Physicochemical Analysis

In situ or immediate laboratory measurements of pH, temperature, turbidity, conductivity, total dissolved solids (TDS), salinity, and dissolved oxygen (DO) were performed on the 500 mL unfiltered aliquot using calibrated multiparameter probes (HQ30d/HQ40d, HACH, USA), a conductivity meter (MM150, HACH, USA), and a portable turbidimeter (2100Q, HACH, USA). Nutrient concentrations, including ammoniacal nitrogen (NH_3_‐N), nitrite nitrogen (NO_2_‐N), and nitrate nitrogen (NO_3_‐N), along with UV_254_ absorbance, were determined from the non‐acidified filtrate (~300 mL) using a UV‐VIS DR‐6000 spectrophotometer (HACH, USA) following standard APHA methods (Rice et al. 2012). Total organic carbon (TOC) was quantified from a 40 mL unfiltered aliquot using a Sievers InnovOx ES laboratory TOC analyzer with an autosampler (Veolia, USA) based on supercritical water oxidation. All instruments were calibrated according to the manufacturer's specifications before analysis.

### 2.4 Metal Analysis by ICP‐OES

For dissolved metal quantification, 200 mL of the 0.45 µm filtrate was acidified to pH < 2 with concentrated HNO_3_ and stored at 4°C until analysis. Concentrations of Fe, Mn, Al, Cu, Zn, and Pb were determined using inductively coupled plasma optical emission spectrometry (ICP‐OES; Avio® 200, PerkinElmer, USA). External calibration was performed using Multi‐Element Standard 3 (PerkinElmer) prepared in 1% HNO_3_. Element‐specific wavelengths were selected to minimize spectral interference.

### 2.5 DNA Extraction

For each replicate, the 1 L aliquot was filtered through 0.22 µm membrane filters to capture microbial biomass. Total DNA was extracted using the PowerWater DNA Isolation Kit (Qiagen) according to the manufacturer's instructions. DNA integrity was assessed by 0.8% agarose gel electrophoresis, and purity was evaluated by measuring the A260/A280 absorbance ratio using a NanoDrop 2000 spectrophotometer (Thermo Scientific, USA). Extracted DNA was stored at −20°C until sequencing.

### 2.6 Library Preparation and Shotgun Metagenomic Sequencing

Metagenomic libraries were prepared using a modified Nextera XT–based (iNextEra) transposase tagmentation protocol. DNA (2.5 μL) was tagmented with bead‐linked transposomes (Illumina DNA Library Prep) and 2 × TMP buffer (Jones et al. 2023) in a total reaction volume of 6 μL, followed by incubation at 53°C for 30 min. Adapter ligation and dual indexing were performed via limited‐cycle PCR using custom iNextEra primers. PCR reactions (62.5 μL total volume) contained Q5 High‐Fidelity DNA Polymerase (New England Biolabs), reaction buffer, dNTPs, and index primers. Cycling conditions included strand repair (72°C, 3 min), initial denaturation (98°C, 3 min), 12 amplification cycles (98°C for 45 s, 62°C for 30 s, 72°C for 2 min), and final extension (72°C, 1 min). Amplified libraries (300–700 bp) were verified by agarose gel electrophoresis, quantified using Qubit Flex (Thermo Fisher Scientific), normalized, pooled, and purified using AMPure XP beads (Beckman Coulter). Paired‐end sequencing (2 × 150 bp) was performed on an Illumina NovaSeq X Plus platform (Novogene, USA). Details regarding sequencing depth and data volume are provided in Supplementary Dataset S1.

### 2.7 Taxonomic Classification and Functional Annotation of Metagenomic Sequences

Initial quality evaluation of raw FASTQ files was conducted using FastQC (v0.11.6) (Andrews 2010). Adapter trimming and quality filtering were carried out with Trimmomatic (v0.39) (Bolger et al. 2014). employing strict parameters: a 4 bp sliding window, a minimum Phred quality score of 20, and a minimum read length of 50 bp. On average, 70.51 million read pairs per sample were retained post‐filtering (range: 52.98–81.91 million pairs). To enrich for microbial sequences, host‐derived reads were filtered out using BBduk from the BBtools package (v38.x) (Bushnell 2014). This step was performed in silico by aligning the quality‐trimmed reads against the human reference genome (GRCh38, GCA_000001405.15_GRCh38_no_alt_analysis_set. fna) using a k‐mer length of 31. Reads that did not align to the host genome were retained as “host‐filtered” and used for downstream taxonomic profiling. Filtered reads were classified taxonomically using Kraken2 (v2.1.x) (Wood et al. 2019) with a confidence threshold of 0.1 and a minimum of 2 hit groups required for classification. The analysis was performed against a custom Kraken2 database, a prebuilt Kraken2 database, last updated in July 2025 (https://benlangmead.github.io/aws-indexes/k2). The database included archaeal, bacterial, viral, and fungal genomes from RefSeq or other standard sources, though the exact composition is user‐defined. The Kraken2 classification reports were subsequently processed with Bracken (Bayesian Re‐estimation of Abundance after Classification with KrakEN) (Lu et al. 2017) to achieve more accurate species‐level abundance estimates. Taxonomic identification was enhanced using TaxonKit (Shen and Ren 2021). Detailed sequencing quality‐control metrics, including trimming efficiency, host‐read removal, and microbial‐read classification statistics, are provided in Dataset S1.

We utilized a read‐based classification approach rather than assembly‐based analysis to maintain compatibility with established resistome profiling pipelines and ensure comprehensive detection of low‐abundance resistance determinants. Nevertheless, the high sequencing depth achieved in this study (Dataset S1) is also suitable for future assembly‐based recovery of metagenome‐assembled genomes (MAGs), which would enable strain‐resolved analyses of resistance gene mobilization and host‐taxon associations.

Functional profiling of antimicrobial resistance was performed using the AMR++ pipeline (Bonin 2023). High‐quality reads were mapped to the MEGARes database (v2.0) (Bonin 2023; Chen et al. 2005). Only gene matches with ≥ 99% sequence coverage were retained to ensure specificity. The resulting high‐confidence gene identifications were analyzed using ResistomeAnalyzer (v2.1; https://github.com/cdeanj/resistomeanalyzer) to characterize antimicrobial resistance profiles. This stringent filtering minimized partial matches and enhanced the accuracy of resistome profiling.

Reads Per Kilobase (RPK) gene family abundance tables from HUMAnN3 (Beghini et al. 2021) were processed to consolidate UniRef90 IDs (Suzek et al. 2015) and remove taxonomic annotations. Gene abundances were summed for each UniRef90 identifier to eliminate redundancy. This pipeline ensured comprehensive metabolic profiling while maintaining compatibility with downstream statistical and comparative analyses (Tenenbaum et al. 2019).

### 2.8 Bioinformatic and Microbial Community Analysis

Microbial diversity analyses were performed in R (v4.2.0) using RStudio (2022.07.1), primarily utilizing the *phyloseq* (v1.50.0) (McMurdie and Holmes 2013) and *vegan* (v2.6‐4) packages (Oksanen et al. 2013). Community composition data at the phylum, class, genus, and species levels were used to compute diversity indices and to visualize relative abundances (Gu et al. 2014; Rout et al. 2024b). Stacked bar plots were generated to compare taxonomic profiles between the two sampling periods (December vs. February). All relative abundance values were derived from shotgun metagenomic sequencing data, and analyses were performed using reproducible scripts within the R statistical environment.

## 3 Results

### 3.1 Water Quality Differences Between Sampling Periods

The two sampling periods represented contrasting environmental conditions in the Shitalakshya River (Table 1). In December 2024, the river water had a pH of 7.4, DO of 2.16 mg/L, turbidity of 18.4 NTU, and moderate nutrient levels (NH_3_‐N: 5.1 mg/L, NO_3_‐N: 0.1 mg/L). By February 2025, pH dropped to 7.0, and DO plummeted to 1.69 mg/L, indicating severe hypoxic conditions. Turbidity increased to 29.4 NTU, while conductivity, TDS, and salinity rose substantially (TDS from 283 to 372 mg/L), suggesting accumulation of suspended solids and dissolved ions as river flow decreased. Nitrogenous compounds increased sharply (NH_3_‐N: 9.95 mg/L, NO_3_‐N: 0.8 mg/L), and TOC roughly doubled from ~7.91 to 15 mg/L. Overall, NH_3_–N increased by 95%, NO_3_‐N by 89%, TOC by 89%, turbidity by 59%, and conductivity by 33%, indicating substantial deterioration in water quality during this period.

**Table 1:** Comparative physicochemical characteristics of Shitalakshya River water during early (December 2024) and peak (February 2025) dry‐season conditions.

Sample collection time	pH	Temp.	Turbidity	Conductivity	TDS	Salinity	D.O.	NH_3_‐N	NO_2_‐N	NO_3_‐N	TOC	UV_254_
		°C	NTU	µS/cm	mg/L	mg/L	mg/L	mg/L	mg/L	mg/L	mg/L	cm^−1^
December 2024	7.4	22.6	18.4	544	283	261	2.16	5.1	0.013	0.1	7.91	0.1128
February 2025	7.0	23.8	29.4	723	372	345	1.69	9.95	0.027	0.8	15	0.1558

### 3.2 Increased Metal Concentrations

**Figure 1:**
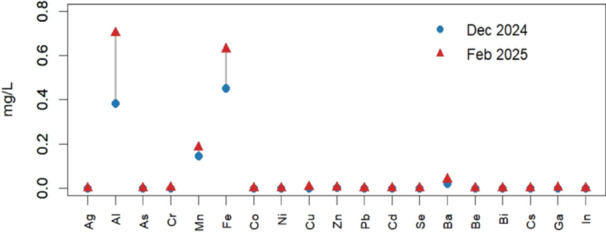
Metal concentrations in Shitalakshya River water during early and peak dry‐season conditions (December 2024 vs. February 2025). Points represent measured concentrations (mg/L) of individual metals in each sampling period, and vertical connecting lines indicate the magnitude and direction of change.

Analysis revealed the presence of multiple trace and heavy metals, with generally higher concentrations in February 2025 than in December 2024 (Figure 1 **).** Aluminum, iron, manganese, chromium, and arsenic showed notable increases. Aluminum concentration rose from 0.384 mg/L to 0.702 mg/L, iron from 0.451 to 0.628 mg/L, and manganese from 0.145 to 0.185 mg/L (Table S1). Chromium increased threefold (0.001 to 0.003 mg/L), and arsenic undetected in December, was measured at 0.001 mg/L in February. Other metals were consistently detected at low concentrations, while cadmium and silver remained below detection limits. These results indicate dry‐season accumulation of metals, consistent with declining dilution capacity and sediment water interactions.

### 3.3 Pronounced Temporal Shifts in Microbial Community Composition

**Figure 2:**
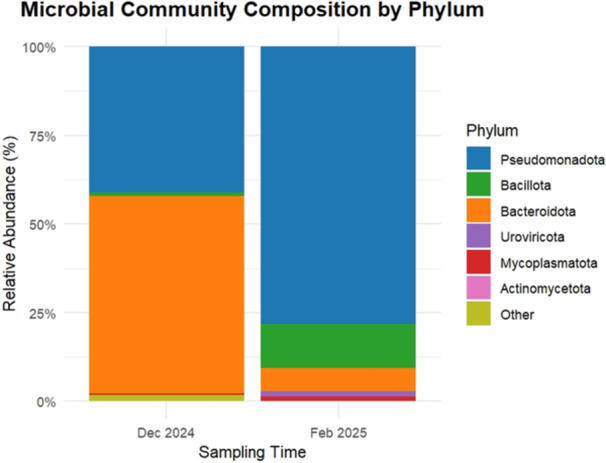
Phylum‐level composition of microbial communities in the Shitalakshya River during early (December 2024) and peak (February 2025) dry‐season conditions. Relative abundances (%) indicate a marked shift from Bacteroidota‐dominated to Pseudomonadota‐dominated communities.

A total of 13 bacterial phyla were identified across the two sampling points (Figure 2; Dataset S1). In December 2024, the community was dominated by Bacteroidota (55.62%) and Pseudomonadota (41.06%). By February 2025, a pronounced restructuring occurred, with Pseudomonadota increasing to 78.19% and Bacteroidota declining sharply to 6.40%. Bacillota increased from ~1% to 12.50%, and minor increases were observed in Uroviricota and Mycoplasmatota. This shift reflects selection for stress‐tolerant, metabolically versatile taxa under hypoxic and pollutant‐enriched conditions. The increased dominance of Pseudomonadota and decline of Bacteroidota are consistent with patterns observed in polluted river systems globally, where facultative and metabolically adaptable taxa are favored under hypoxic and chemically stressed environments (Behera et al. 2020a; Premke et al. 2020).

**Figure 3:**
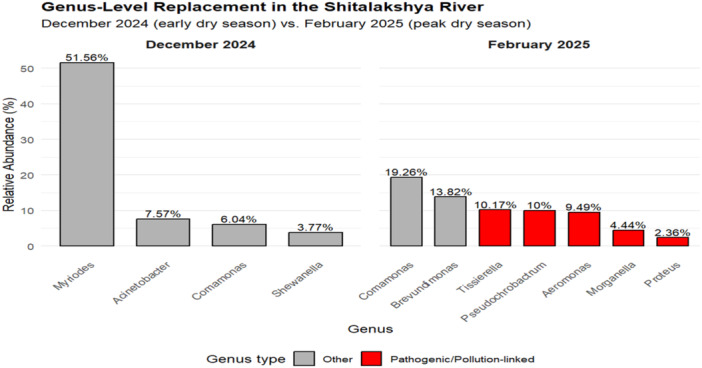
Genus‐level distribution of dominant microbial taxa in the Shitalakshya River showing a shift from Myroides dominance to a more evenly distributed community with enrichment of *Comamonas*, *Brevundimonas*, *Tissierella*, and *Aeromonas*, indicating stress‐driven community restructuring.

At the genus level, December 2024 samples were strongly dominated by *Myroides* (51.56%), followed by *Acinetobacter* (7.57%), *Comamonas* (6.04%), and *Shewanella* (3.77%) (Figure 3; Dataset S1). In February 2025, community structure became markedly more even, with enrichment of *Comamonas* (19.26%), *Brevundimonas* (13.82%), *Tissierella* (10.17%), and *Pseudochrobactrum* (10.00%). Opportunistic and pollution‐associated genera, including *Aeromonas* (9.49%), *Morganella* (4.44%), and *Proteus* (2.36%), increased substantially.

**Figure 4:**
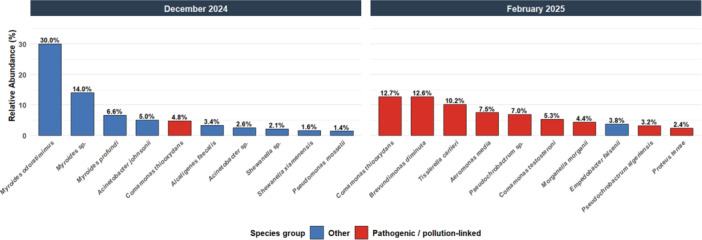
Species‐level microbial composition across two dry‐season time points showing transition from *Myroides‐dominated* to diverse stress‐adapted community, with no single species exceeding 15% relative abundance.

Species‐level analysis reinforced this restructuring. December communities were highly skewed toward *Myroides odoratimimus* (29.96%), *Myroides sp*. (14.03%), and *Myroides profundi* (6.58%). In contrast, February samples showed distributed dominance among Comamonas thiooxydans (12.66%), Brevundimonas diminuta (12.65%), Tissierella carlieri (10.17%), Aeromonas media (7.50%), and Pseudochrobactrum spp. (6.96%), with no single species exceeding 15% abundance (Figure 4; Dataset S1).

This transition indicates replacement of a Myroides‐dominated assemblage by a more functionally diverse but stress‐adapted community. The enrichment of facultative anaerobic and opportunistic taxa under hypoxic, nutrient‐rich conditions aligns with ecological theory predicting deterministic environmental filtering in stressed ecosystems.

### 3.4 Seasonal Restructuring of the Resistome

**Figure 5:**
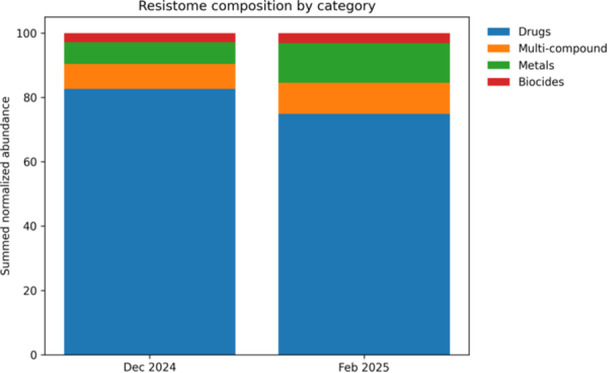
Resistome composition of the Shitalakshya River under contrasting dry‐season conditions, showing shifts in resistance categories. A slight reduction in ARGs is observed alongside enrichment of metal and multi‐compound resistance in the peak dry season.

Both metagenomes harbored a dense and diverse resistome encompassing resistance to antibiotics, metals, biocides, and multi‐compound stressors (Figure 5; Dataset S1). While drug/antibiotic resistance genes (ARGs) remained dominant at both time points, their relative abundance declined from 86.6% in December 2024 to 76.9% in February 2025. In contrast, metal resistance genes (MRGs) increased from 6.8% to 12.25%, and biocide resistance genes (BRGs) increased modestly (2.8% to 3.2%). Multi‐compound resistance genes showed a slight decline from 3.7% to 2.7%.

### 3.5 Antibiotic Resistance Gene (ARG) Landscape

**Figure 6:**
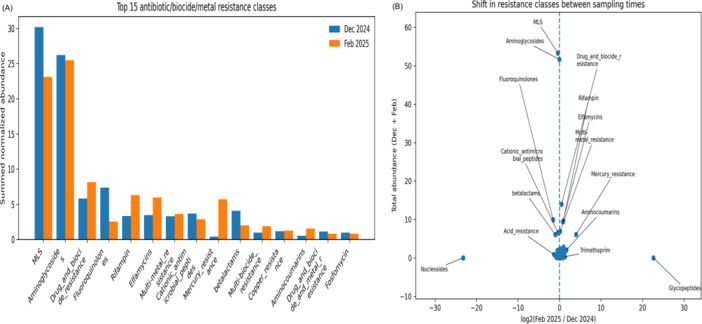
Antibiotic, biocide, and metal resistance gene dynamics across early and peak dry‐season conditions. (A) Relative abundance of the top 15 resistance classes detected in December 2024 and February 2025 samples. MLS, multidrug, and aminoglycoside resistance genes were dominant in both periods, while rifampicin, elfamycin, and metal resistance classes showed marked enrichment in the peak dry season. (B) Log_2_ fold change in resistance class abundance between sampling periods. Positive values (blue) indicate enrichment in February 2025; negative values (red) indicate depletion. Metal‐associated and multi‐compound resistance classes (mercury, multi‐metal, drug–biocide) are markedly enriched, while nucleoside and glycopeptide resistance classes decline. The dashed line indicates no change.

Shotgun metagenomic profiling detected antibiotic resistance genes (ARGs) spanning 18 distinct antibiotic classes across both sampling periods. Resistance determinants targeting macrolide‐lincosamide‐streptogramin (MLS) and aminoglycosides dominated the resistome, collectively accounting for approximately 56% of total ARG abundance in December 2024 (MLS ~ 30%; aminoglycosides ~26%) (Figure 6). This dominance was primarily driven by ribosomal target modification genes, notably MLS23S (26.14%) and A16S (18.43%), together with macrolide efflux pumps (e.g., *msrE*, *mecF*), which comprised nearly half of the total ARG burden. Resistance to rifampicin (*rpoB*, 3.32%) and elfamycins (*tufAB*, 3.45%) was consistently detected across replicates (Table S2). β‐Lactam and fluoroquinolone resistance was distributed among multiple low‐abundance determinants, including β‐lactamase genes (*bla*CTX, *bla*OXA, *bla*TEM), chromosomal mutations (*gyrA*, *gyrB*), and plasmid‐mediated fluoroquinolone protection genes (*qnrD*, 5.90%). Resistance genes for tetracyclines, sulfonamides, phenicols, glycopeptides, and trimethoprim were detected at comparatively low relative abundances (< 1%). Notably, fragments of last‐resort resistance determinants, including *mcr*‐type colistin resistance genes and NDM‐like carbapenemase genes, were detected at very low abundance (< 0.1%).

By February 2025, overall ARG relative abundance declined by approximately 11%; however, the resistome underwent clear compositional restructuring (Figure 6). While A16S remained relatively stable (18.50%), MLS23S declined substantially to 18.80%. Concurrently, rifampicin and elfamycin resistance determinants expanded markedly: *rpoB* nearly doubled from 3.32% to 6.29%, and *tufAB* increased from 3.45% to 5.98% (Figure 6). Several metal‐associated and multi‐compound resistance classes, including mercury, multi‐metal, and drug–biocide resistance, showed notable enrichment in the peak dry season, whereas nucleoside and glycopeptide resistance classes declined. Plasmid‐mediated fluoroquinolone resistance genes (*qnrD*) also decreased, and last‐resort resistance determinants remained detectable but rare (< 0.1%) with no evidence of seasonal amplification. Collectively, these results indicate that dry‐season progression drives selective redistribution of ARG classes rather than simple attenuation of total ARG abundance, with non‐antibiotic resistance determinants contributing to the restructuring of the resistome under increasing pollution stress.

### 3.6 Metal Resistance: Strong Enrichment of Mercury and Multi‐Metal Efflux Systems

**Figure 7:**
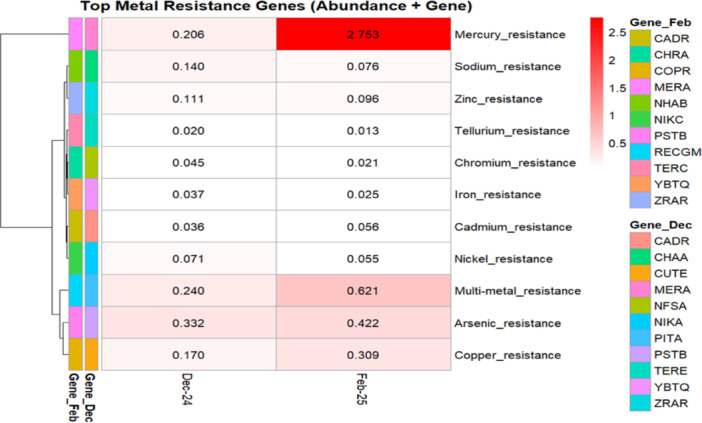
Heatmap showing temporal dynamics of metal resistance gene abundance across early and peak dry‐season samples. Normalized abundances are displayed, with hierarchical clustering revealing similarity patterns among resistance categories. Mercury resistance genes exhibit a pronounced increase in the peak dry season, while multi‐metal, arsenic, and copper resistance genes also show enrichment, reflecting heightened metal‐driven selective pressure. Colored side annotations denote the dominant gene families associated with each resistance category.

In contrast to the slight decline in total ARG abundance, MRGs showed marked amplification in February 2025. Mercury resistance exhibited the largest relative increase, driven by expansion of canonical *mer* operon genes (*merA*, *merT*, *merP*, *merR*, *merD*) (Figure 7).

Multi‐metal resistance genes associated with RND and ABC‐type efflux systems (*cusABC*, *silABC*, *modABC*) were also enriched, indicating enhanced broad‐spectrum metal tolerance. While some resistance classes (e.g., sodium, chromium) declined, the overall profile shifted toward mercury detoxification and multi‐metal efflux dominance, consistent with elevated dissolved metal concentrations measured during the late dry season.

### 3.7 Temporal Activation of Multi‐Compound Efflux Systems

Metagenomic‐based functional profiling revealed selective restructuring of multi‐compound resistance gene expression between December 2024 and February 2025. Among 85 genes representing 10 resistance mechanisms (RND, ABC, MFS, SMR, MATE, and associated regulators), 24 increased (10 newly induced), 17 decreased, and 44 remained stable, indicating targeted activation rather than global upregulation. RND‐type efflux pumps showed the strongest induction. *mexK* was undetectable in December but highly expressed in February (0.893), while *tetB* and *mexD1* more than doubled (Supplementary Table S3). Additional RND‐associated genes (*mexF*, *sme*, *sdeB*, *sdeY*) were newly detected, indicating coordinated activation of multidrug efflux pathways. Regulatory shifts supported this pattern, with *smeT* and *yajC* induced and *mexT* repressed. In contrast, ABC, MFS, and SMR families exhibited heterogeneous responses: *sitABCD*, *fetA*, and *fetB* declined, whereas MFS genes (*emrA*, *emrB*) were newly expressed; *qacEΔ1* increased markedly while *qacF* was repressed. The resistance‐associated protein *ruvB* doubled in expression, and the metal resistance gene *chr* increased by ~173% (0.026 to 0.071), with induction of *chrB*, linking efflux activation to metal‐responsive pathways.

**Figure 8:**
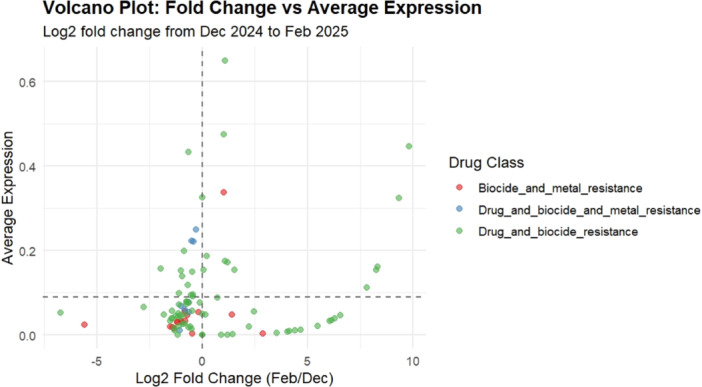
Volcano plot showing log_2_ fold change versus average expression of resistance genes between sampling periods. Each point represents a resistance gene, with positive values indicating enrichment and negative values indicating reduction in the later sampling period. Genes associated with drug–biocide resistance are predominantly enriched, while biocide–metal resistance genes show comparatively limited variation. Dashed lines denote thresholds for fold change and expression.

Volcano plot analysis (Figure 8) confirmed class‐specific transcriptional shifts, with drug‐ and biocide‐associated genes showing the strongest positive log_2_ fold changes. Overall, late dry‐season conditions were characterized by coordinated activation of RND‐dominated multidrug efflux networks, consistent with intensified combined metal and chemical stress.

### 3.8 Biocide Resistance Gene Profiles and Seasonal Shifts

**Figure 9:**
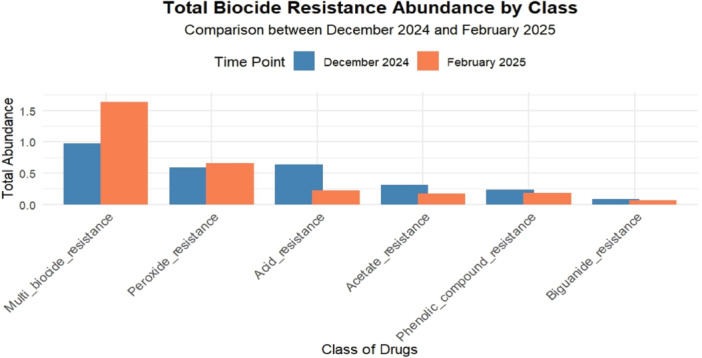
Comparison of total biocide resistance gene abundance between December 2024 and February 2025. The bar chart displays the total abundance of biocide resistance genes across six major drug classes. The classes are arranged in descending order of total abundance. A notable significant increase in multi‐biocide resistance genes (Multi_biocide_resistance) was observed in February 2025 compared to the previous sampling period. Conversely, levels of acid resistance (Acid_resistance) and acetate resistance (Acetate_resistance) decreased during the same timeframe.

A diverse repertoire of biocide resistance genes was detected across both sampling periods, encompassing acetate, acid, biguanide, peroxide, phenolic compound, and multi‐biocide resistance mechanisms. Overall, the resistome was dominated by multi‐biocide resistance determinants and oxidative stress‐associated genes. Metagenomic analysis identified a diverse repertoire of biocide resistance genes (BRGs) across both sampling periods, including determinants associated with resistance to acetate, acids, biguanides, peroxides, phenolic compounds, and multi‐biocide stressors (Figure 9).

Biocide resistance profiles underwent clear restructuring from December to February, paralleling shifts observed in antibiotic and metal resistance categories **(**Supplementary Table S4 **).** In December 2024, *sodB* (peroxide resistance) was the most abundant BRG (0.3260), followed by *yjcG* (0.2906) and *lpdT* (acetate resistance; 0.2603). By February 2025, the efflux‐associated multi‐biocide resistance gene *mexW, which was* undetectable in December, became the dominant BRG (0.5826), representing the largest temporal increase observed. Moderate enrichment was also recorded for *sodB* and *yjcG*, whereas *lpdT*, *rpoS*, *fabG*, and *cepAB* declined. Efflux‐associated and global stress regulators such as *tolC*, *kexD*, and *oxyR* remained comparatively stable. The strong induction of *mexW*, together with concurrent enrichment of RND‐type multidrug efflux systems in February, indicates coordinated activation of broad‐specificity transport networks under intensified dry‐season stress. Given that such efflux platforms frequently mediate tolerance to antibiotics, metals, and disinfectants, the observed BRG restructuring further supports a co‐selection framework in which non‐antibiotic stressors promote the maintenance of multidrug‐resistance architectures within the riverine microbial community.

### 3.9 Novel Integrative Findings: Environmental Stress as an Ecological Bottleneck

Unlike previous river‐based metagenomic studies that primarily describe ARG inventories or broad seasonal contrasts, this study demonstrates that dry‐season intensification alone can drive rapid restructuring of the microbiome and resistome in a heavily polluted, climate‐stressed tropical river. The combined observation of (i) collapse of *Myroides* dominance, (ii) enrichment of opportunistic and facultative anaerobic genera, and (iii) strong selection for mercury resistance and multi‐compound RND efflux systems indicates that late dry‐season conditions act as a selective bottleneck favoring multidrug‐tolerant assemblages. The concurrent increase in nutrients, TOC, and metals alongside resistome restructuring suggests strong environmental coupling. Rather than antibiotic input alone, combined hypoxia, nutrient loading, and metal stress appear to function as dominant drivers shaping multidrug resistance potential. This ecological filtering interpretation aligns with niche‐based community assembly theory, where environmental stressors select for organisms with pre‐adapted tolerance mechanisms (Galand et al. 2016).

## 4 Discussion

This study provides the first targeted metagenomic evidence of microbial community and resistome restructuring in the Shitalakshya River under contrasting dry‐season conditions. The marked water quality deterioration observed during the peak dry season characterized by hypoxia, elevated nutrient concentrations, and increased organic carbon and metal loads, coincided with pronounced shifts in microbial community composition and resistance gene profiles.

### 4.1 Community Restructuring as Deterministic Environmental Filtering

The transition from a *Myroides*‐dominated assemblage (~40% relative abundance in December) to a community enriched with *Comamonas*, *Brevundimonas*, *Tissierella*, and *Aeromonas* in February exemplifies species sorting theory, where environmental stressors act as selective filters eliminating maladapted taxa while favoring stress‐tolerant specialists (Leibold et al. 2004; Holyoak et al. 2005). The near‐complete collapse of *Myroides* spp. copiotrophic r‐strategists lacking specialized xenobiotic degradation pathways and the concurrent expansion of metabolically versatile taxa demonstrates deterministic exclusion driven by intensified chemical stress (Grime 1977; McGill et al. 2006). This shift from r‐selected to S‐selected life history strategies is consistent with patterns observed in polluted river systems globally (Behera et al. 2023; Premke et al. 2020; Rout et al. 2022, 2023, 2024a).

The specific metabolic capabilities of enriched taxa underscore the functional significance of this restructuring. *Comamonas thiooxydans* and *C. testosteroni* (combined ~18% relative abundance) harbor pathways for aromatic hydrocarbon degradation, including PAHs and PCBs (Jiang et al. 2023a), while the 28.7‐fold increase in *Brevundimonas diminuta* is linked to its heavy metal resistance and pesticide degradation capacity (Ryan and Pembroke 2018). The emergence of *Pseudochrobactrum* spp. (289,624 → 1,988,304 reads), known chromate‐reducing specialists, further indicates that specific contaminant classes rather than general organic enrichment structured the February assemblage (Knight et al. 2018).

Beyond dominant taxa, several less abundant genera exhibited coordinated enrichment patterns that strengthen the causal linkage between taxonomy and contaminant degradation. A 3.7‐fold increase in *Rhodoferax* spp. indicates their role in coupling heavy‐metal reduction with organic contaminant oxidation. This increase highlights a key bioremediation mechanism. As seen in *R. ferrireducens*, these bacteria oxidize organic pollutants and transfer electrons to heavy metals, immobilizing them (Risso et al. 2009). This metabolic versatility is fundamental to carbon and metal cycling in subsurface environments and aligns with the organism's novel ability to convert chemical energy into electricity. The increase in *Curvibacter* reads from 43 to 105 during the peak dry season suggests its functional importance in degrading persistent organic pollutants like phthalate esters under the stressed, low‐nutrient conditions (Ma et al. 2016). The increase in *Azospira* reads from 187 to 626 during the dry season suggests its functional role in nitrate and perchlorate reduction, as this genus is known to preferentially reduce these co‐contaminants under the anaerobic conditions typical of highly polluted river water (Nam et al. 2016). *Achromobacter xylosoxidans* (6.0‐fold increase) degrades halogenated aromatics and organophosphate pesticides, while *Alicycliphilus denitrificans* (2.8‐fold increase) performs anaerobic aromatic hydrocarbon degradation coupled to denitrification. This coordinated enrichment reflects deterministic selection for a functionally integrated assemblage wherein aerobic degraders (*Comamonas*, *Pseudomonas*, *Achromobacter*) initiate contaminant transformation, facultative anaerobes (*Alicycliphilus*, *Shewanella*) continue degradation using alternative electron acceptors, and strict anaerobes (*Dehalobacter*, *Tissierella*) complete mineralization through reductive dehalogenation (DeAngelis et al. 2010; Pett‐Ridge and Firestone 2005).

This cascading metabolic architecture was spatially organized along a compressed redox gradient, evidenced by the co‐occurrence of obligate aerobes, facultative anaerobes (*Aeromonas media*, 12,596 → 1,470,792 reads), and strict anaerobes (*Tissierella carlieri*, 0 → 1,995,804 reads). The February community exhibited characteristics of a stress‐driven cooperative network, with syntrophic linkages between fermentative bacteria (*Proteiniborus*, *Sedimentibacter*) and organohalide‐respiring taxa (*Dehalobacter*, *Dehalobacterium*) consistent with predictions of the stress gradient hypothesis (Hernandez et al. 2021; Hoek et al. 2016). *Comamonas* spp. likely function as keystone degraders, remodeling the community to enhance cooperative degradation networks (Jiang et al. 2023b).

The enrichment of opportunistic genera, including *Aeromonas*, *Acinetobacter*, and *Morganella*, highlights potential public health implications for drinking‐water abstraction (LeChevallier et al. 2024; Sharif et al. 2025). Within the source‐sink framework, clinically relevant taxa such as *Klebsiella pneumoniae* and *Pseudomonas aeruginosa* likely exist as sink populations maintained by continuous allochthonous input from anthropogenic sources rather than in situ proliferation (Pulliam 1988).

### 4.2 Redox Gradients and Syntrophy as Drivers of Degradation

Functional complementarity was structured along a compressed redox gradient, evidenced by the co‐occurrence of obligate aerobes (*Comamonas*), facultative anaerobes (*Aeromonas media*, *Shewanella*), and strict anaerobes (*Tissierella carlieri*, *Alkaliphilus oremlandii*). This vertical stratification supports redox niche theory, where sequential anaerobic–aerobic metabolism enables complete contaminant mineralization across microzones. The expansion of strict anaerobes in the late dry season indicates intensified sediment oxygen demand and a compressed aerobic zone.

The February community displayed characteristics of a stress‐driven cooperative network rather than isolated populations. In line with the stress gradient hypothesis, positive interactions (syntrophy) prevailed under high contaminant stress. Evidence for syntrophic coupling included linkages between fermentative bacteria (*Proteiniborus*, *Sedimentibacter*, *Lacrimispora*) producing H_2_ and acetate and organohalide‐respiring taxa (*Dehalobacter*, *Dehalobacterium formicoaceticum*) requiring these substrates (Zhu et al. 2020). This network reflects ecological facilitation cascades, with *Comamonas thiooxydans* and *C. testosteroni* acting as keystone degraders that remodel the community to enhance cooperative degradation networks (Altieri et al. 2007).

### 4.3 Turbidity and Particulate‐Associated Community Assembly

Turbidity, a proxy for suspended particulate matter (Serajuddin et al. 2019b), showed a marked increase and likely serves as a dominant environmental factor shaping these microbial shifts by providing surfaces for biofilm formation and particle‐attached bacterial growth (Adhikari et al. 2025). However, the relative importance of turbidity versus other correlated variables (e.g., TOC, metals) requires further investigation through multivariate approaches in future studies with expanded sampling designs.

### 4.4 Metal‐Driven Co‐Selection as an Ecological Filter for the Resistome

The resistome analysis revealed a complex and dynamic resistance landscape. Although total ARG abundance decreased from 86.6% to 76.9% during the peak dry season, the 2.2‐fold enrichment of metal resistance genes (MRGs, 6.8% to 12.25%) particularly the pronounced amplification of mercury resistance determinants (*merA*, *merT*, *merP*, *merR*, *merD*) provides compelling evidence that metal stress functions as a primary ecological filter structuring the late dry‐season resistome. The concurrent 2.7‐fold increase in aluminum (0.384 to 0.702 mg/L) and threefold increase in chromium (0.001 to 0.003 mg/L) concentrations measured during February establish a direct causal linkage: elevated metal bioavailability imposed selective pressure favoring taxa harboring constitutive metal detoxification systems (Gillieatt and Coleman 2024; Engin et al. 2023). These patterns are consistent with previous studies demonstrating that heavy metals and industrial contaminants can promote co‐selection of antimicrobial resistance by maintaining linked resistance determinants within microbial communities (Chukwu et al. 2023; Engin et al. 2023; Gillieatt and Coleman 2024).

The localization of *mer* operons on broad‐host‐range mobile genetic elements transforms metal resistance into a community‐level property, enabling positive selection across phylogenetically diverse lineages from *Pseudomonadota* to *Bacillota* (Chukwu et al. 2023). This interpretation is reinforced by parallel enrichment of multi‐metal RND‐type efflux systems (*cusABC*, *silABC*). In such environments, non‐antibiotic stressors may play an important role in sustaining multidrug resistance even in the absence of increased antibiotic inputs. Similar co‐selection dynamics have been documented in other South Asian rivers receiving industrial effluents (Faruk et al. 2025; Rout et al. 2022, 2023, 2025), reinforcing the relevance of these mechanisms in the regional context.

### 4.5 Efflux Pump Activation and Cross‐Resistance Networks

The coordinated activation of RND‐type multidrug efflux systems during February—induction of *mexK* (undetectable to highly expressed), doubling of *tetB* and *mexD1*, and strong upregulation of *mexW* (undetectable to dominant, 0.5826)—represents a critical mechanistic link between environmental stress and antimicrobial resistance potential. RND efflux pumps accommodate antibiotics, metals, biocides, and organic solvents within overlapping binding pockets, conferring cross‐resistance to structurally unrelated compounds (Gillieatt and Coleman 2024). The selective induction of specific RND genes rather than uniform upregulation, coupled with metal‐responsive regulator activation (*smeT* induction, *mexT* repression), suggests fine‐tuned environmental sensing rather than generalized stress response, creating an ecological memory wherein historical metal exposure maintains multidrug resistance potential (Knight et al. 2018).

### 4.6 Resistome Restructuring as Functional Niche Partitioning

The compositional restructuring of ARGs—enrichment of rifampicin (*rpoB*, 3.32% to 6.29%) and elfamycin (*tufAB*, 3.45% to 5.98%) resistance alongside declines in MLS resistance indicates functional niche partitioning under species sorting. The paradoxical enrichment of *rpoB* mutations, despite their documented fitness costs, can be explained by pleiotropic effects enhancing solvent stress and oxidative damage tolerance under combined chemical stress (Cutugno et al. 2020; Faruk et al. 2025). Last‐resort resistance determinants (*mcr*‐type, NDM‐like) remained at consistently low abundances (< 0.1%) without seasonal amplification, indicating they exist as sink populations maintained by allochthonous input rather than active in situ selection (Pulliam 1988). Similar co‐selection dynamics have been documented in other South Asian rivers receiving industrial effluents (Faruk et al. 2025; Rout et al. 2022, 2023, 2024a, 2025).

### 4.7 Synthesis: A Trait‐Based Model of Resistome Assembly

Integrating taxonomic and resistome data within Grime's CSR framework, we propose a trait‐based model of resistome assembly under dry‐season contaminant stress. The transition from r‐selected (copiotrophic, fast‐growing) to S‐selected (stress‐tolerant) life history strategies is consistent with CSR triangle theory (Grime 1977, 2001) and its demonstrated applicability to microbial communities under heavy metal and antibiotic selection pressures (Wood et al. 2018; Sharma et al. 2023). This model is reflected in the contrasting resistome configurations observed across seasons: the December resistome exemplifies an r‐selected configuration maintained by high biomass turnover and horizontal gene transfer, while the February resistome represents an S‐selected architecture characterized by lower ARG diversity but enrichment of robust cross‐tolerance mechanisms, including mercury detoxification, RND efflux, and *rpoB* mutations. This resistome shift mirrors the taxonomic transition from *Myroides* (r‐strategist: rapid growth on labile organic matter) to S‐strategists such as *Comamonas*, *Brevundimonas*, and *Pseudochrobactrum* spp., which exhibit constitutive expression of detoxification enzymes, metal efflux pumps, and low maximum growth rates with high substrate affinity.

This convergence demonstrates that intensified environmental stress filters for stress‐tolerant functional traits rather than maximum growth potential (Bertness and Callaway 1994; Sthultz et al. 2007). Critically, this model predicts that resistome composition is driven primarily by non‐antibiotic stressors (metals, organic pollutants, hypoxia) rather than antibiotic concentrations alone, suggesting that controlling industrial metal discharges may be equally important as antibiotic stewardship for AMR mitigation in urban river systems (World Health Organization 2015).

### 4.8 Implications for Public Health and Water Safety

The enrichment of opportunistic pathogens, including *Aeromonas*, *Acinetobacter*, and *Morganella*, under peak pollution conditions raises concerns for drinking‐water safety in abstraction‐dependent systems (LeChevallier et al. 2024; Sharif et al. 2025). Clinically relevant taxa such as *Klebsiella pneumoniae*, *Acinetobacter baumannii*, and *Pseudomonas aeruginosa* were detected at low but consistent abundances, suggesting they persist as sink populations maintained by continuous allochthonous input from anthropogenic sources rather than in situ proliferation (Pulliam 1988; Leibold et al. 2004). Their increased detection in February, for example, *Bacteroides thetaiotaomicron* (65 → 16,126 reads) and *Clostridium tetani* (67 → 11,490 reads) likely reflects concentrated fecal inputs during reduced flow, consistent with evaporative concentration effects during the late dry season. While viability and infectivity were not assessed, these enrichment patterns merit inclusion in future risk‐based monitoring programs.

### 4.9 Practical Implications for Bioremediation and Water Safety

Practical implications for bioremediation and water safety include using stress‐tolerant genera and resistance genes as microbial indicators of pollution. Metal‐driven co‐selection of antimicrobial resistance underscores the need to control industrial metal discharges alongside antibiotics. Remediation must address combined chemical stressors, not single pollutants. From a metacommunity perspective, the February community with established cooperative metabolic networks may resist displacement even after monsoon dilution, representing a persistent degraded‐state community exhibiting hysteresis (Scheffer et al. 2001; Beisner et al. 2003). This has significant implications for bioremediation, as natural attenuation may prove insufficient to restore pre‐contamination community structure. Integrating metagenomic surveillance into routine water quality monitoring would enable early detection of resistome shifts and inform interventions before critical thresholds are exceeded (World Health Organization 2015).

### 4.10 Conceptual Framework

**Figure 10:**
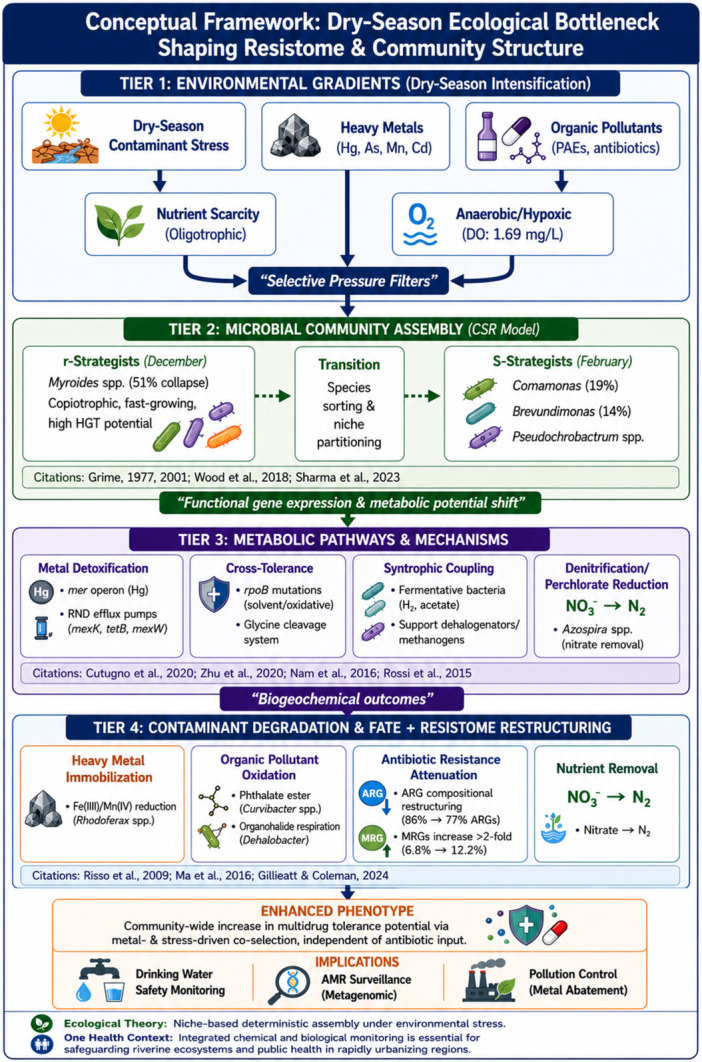
Conceptual framework illustrating how dry‐season environmental stressors drive deterministic microbial community assembly, metabolic pathway shifts, and resistome restructuring through metal‐ and stress‐driven co‐selection in the Shitalakshya River, with implications for antimicrobial resistance and drinking water safety.

Climate‐driven water scarcity concentrates anthropogenic pollutants (heavy metals, nutrients, organic carbon), creating a selective ecological bottleneck (Figure 10). This deterministic environmental filtering drives a taxonomic shift characterized by the collapse of sensitive taxa (e.g., *Myroides* spp. [51%]) and the enrichment of stress‐tolerant opportunistic genera (*Comamonas* [19%], *Aeromonas* [9%], *Brevundimonas* [14%]). Concurrently, the resistome undergoes restructuring: while antibiotic resistance genes (ARGs) show a modest decline (86% to 77%), metal resistance genes (MRGs) increase > 2‐fold (6.8% to 12.2%). This shift is driven by metal‐driven co‐selection and stress adaptation, activating efflux pumps (*mexK/tetB*) and biocide resistance (*mexW*) via mobile genetic elements. The resulting community‐wide enhancement of multidrug tolerance potential occurs independently of clinical antibiotic input, posing a direct threat to drinking water safety and public health. The framework underscores the need for integrated chemical (metal abatement) and biological (AMR surveillance) monitoring under the One Health paradigm.

### 4.11 Limitations

This study's restricted sampling design, with two time points at a single site, limits broader generalization and precludes robust statistical biomarker identification using approaches such as LEfSe or DESeq. 2. However, the selected periods represent hydrological extremes of the dry season, providing a focused, high‐contrast comparison of pollution‐driven conditions, an approach employed in other environmental AMR studies for baseline establishment and hypothesis generation (Lu and Lu 2020). Triplicate samples collected on a single day per time point ensure analytical reproducibility but do not capture short‐term temporal variability. Future studies should incorporate multi‐day sampling, expanded spatial coverage, and sediment analysis to assess benthic reservoirs of resistance determinants.

We do not claim that this two‐time‐point design can resolve causal relationships or identify robust biomarkers. Rather, this work generates a testable hypothesis that dry‐season pollutant concentration drives resistome restructuring via metal co‐selection, which now requires validation through expanded spatial, temporal, and sediment sampling. Despite these constraints, the consistency and magnitude of observed shifts across independent data types, physicochemical parameters, multi‐rank taxonomic profiles, and multiple resistance gene categories indicate strong environmental filtering effects that merit further investigation.

## 5 Conclusion

This study demonstrates that intensified dry‐season conditions in a heavily polluted urban river are associated with substantial restructuring of microbial communities and resistome composition. Worsening hydrochemical conditions, including hypoxia, nutrient enrichment, and elevated metal concentrations, coincided with a shift from a *Myroides‐*dominated community to a more diverse assemblage of stress‐tolerant and opportunistic taxa. Concurrently, the resistome exhibited a transition characterized by reduced relative abundance of antibiotic resistance genes and increased representation of metal resistance genes and multidrug efflux systems. These patterns are consistent with the influence of non‐antibiotic stressors in shaping resistance dynamics through co‐selection mechanisms. Although based on limited sampling, this study provides initial metagenomic evidence of microbiome and resistome restructuring in the Shitalakshya River under dry‐season stress. The findings underscore the need for integrated monitoring of microbial and chemical stressors to better understand and manage antimicrobial resistance risks in surface waters used for drinking water supply in rapidly urbanizing regions.

## Author Contributions

**Muhammad Ehteshamul Haque:** investigation, writing – original draft, formal analysis, methodology. **Md. Shaminur Rahman:** methodology, writing – review and editing. **Munawar Sultana:** validation, writing – review and editing. **Anowara Begum:** conceptualization, writing – review and editing, supervision.

## Funding

The authors have nothing to report.

## Ethics Statement

This article does not involve any animal and human objects, only environmental samples.

## Conflicts of Interest

The authors declare no conflicts of interest.

## Supporting information

Supporting File 1.

Supporting File 2.

Supporting File 3.

## Data Availability

The datasets generated during this study are available in the NCBI SRA repository under BioProject PRJNA1424261 (https://www.ncbi.nlm.nih.gov/bioproject/PRJNA1424261) (Accession: SAMN55380971 and SAMN55380975); Dataset S1 includes resistome profiles (ARGs, BRGs, MRGs, MCRGs) and sequencing quality metrics (trimming, host filtering, and Kraken2‐based microbial classification).
