# Supplementary material for: Seasonal Restructuring of Microbial Communities and Resistomes in the Shitalakshya River, Bangladesh Revealed by Shotgun Metagenomics
Source: Microbiologyopen. 2026 Jul 1;15(4):e70359. doi: 10.1002/mbo3.70359 (PMC13322659; doi:10.1002/mbo3.70359)
Supplement: Supplementary file 2 — Supporting File 2. [file MBO3-15-e70359-s002.docx]

**Supplementary Table S1:**

| **Metals (mg/L)** | **December 2024** | **February 2025** |
| --- | --- | --- |
| Aluminum (Al) | 0.384 | 0.702 |
| Iron (Fe) | 0.451 | 0.628 |
| Manganese (Mn) | 0.145 | 0.185 |
| Barium (Ba) | 0.020 | 0.040 |
| Zinc (Zn) | 0.004 | 0.004 |
| Copper (Cu) | 0.001 | 0.006 |
| Chromium (Cr) | 0.001 | 0.003 |
| Nickel (Ni) | 0.001 | 0.000 |
| Cobalt (Co) | 0.001 | 0.001 |
| Arsenic (As) | 0.003 | 0.001 |
| Lead (Pb) | 0.001 | 0.001 |
| Selenium (Se) | 0.004 | 0.007 |
| Gallium (Ga) | 0.001 | 0.004 |
| Bismuth (Bi) | 0.001 | 0.001 |
| **Below detection limit:** Beryllium (Be), Cadmium (Cd), Cesium (Cs), Indium (In), and Silver (Ag) were below the method detection limit (0.000 mg/L) in both sampling periods. | | |

**Supplementary Table S1: Heavy metal concentrations in the Shitalakshya River during December 2024 and February 2025.** Concentrations (mg/L) of dissolved metals measured at the drinking-water abstraction point during late post-monsoon (December 2024) and peak dry season (February 2025).

**Supplementary Table S2:**

| **Rank** | **Drug Class** | **Mechanism** | **Gene** | **Dec.**  **2024** | **Feb.**  **2025** | **Change** |
| --- | --- | --- | --- | --- | --- | --- |
| 1 | Aminoglycosides | Aminoglycoside-resistant 16S ribosomal subunit protein | *A16S* | 18.4278 | 18.5020 | +0.0742 |
| 2 | MLS | Macrolide-resistant 23S rRNA mutation | *MLS23S* | 26.1358 | 18.8015 | ‑7.3343 |
| 3 | Rifampin | Rifampin-resistant beta‑subunit of RNA polymerase RpoB | *rpoB* | 3.3208 | 6.2934 | +2.9726 |
| 4 | Elfamycins | EF‑Tu inhibition | *tufA or tufB)* | 3.4510 | 5.9812 | +2.5302 |
| 5 | Fluoroquinolones | Quinolone resistance protein Qnr | *qnrD* | 5.9015 | 0.7796 | ‑5.1219 |
| 6 | Phenicol | Phenicol resistance MFS efflux pumps | *floR* | 0.0588 | 0.5597 | +0.5009 |
| 7 | betalactams | Class A betalactamases | *blaCTX-M* | 0.7115 | 0.5485 | –0.1631 |
| 8 | betalactams | Class D beta lactamases | *blaOXA* | 0.2763 | 0.4184 | +0.1421 |
| 9 | Aminocoumarins | Aminocoumarin-resistant DNA topoisomerases | *parE* | 0.1921 | 1.2994 | +1.1073 |
| 10 | MLS | MLS resistance ABC efflux pumps | *msrE* | 1.4925 | 1.3169 | –0.1756 |
| 11 | MLS | MLS resistance MFS efflux pumps | *mecF* | 0.8758 | 1.0165 | +0.1407 |
| 12 | Fluoroquinolones | Fluoroquinolone-resistant DNA topoisomerases | *gyrB* | 0.5429 | 1.0005 | +0.4576 |
| 13 | Phenicol | Chloramphenicol acetyltransferases | *catB* | 0.3804 | 0.5028 | +0.1224 |
| 14 | Aminoglycosides | Aminoglycoside O‑nucleotidyltransferases | *ANT3‑DPRIME* | 0.0856 | 0.4067 | +0.3211 |
| 15 | Tetracyclines | Tetracycline resistance MFS efflux pumps | *tetG* | 0.0208 | 0.3601 | +0.3393 |
| 16 | Sulfonamides | Sulfonamide-resistant dihydropteroate synthases | *sul2* | 0.2.214 | 0.3678 | +0.1464 |
| 17 | Beta lactams | Class C beta lactamases | *blaMOX* | 0.0789 | 0.1599 | +0.0810 |
| 18 | Tetracyclines | Tetracycline resistance ribosomal protection proteins | *tetT* | 0.0000 | 0.2165 | +0.2165 |
| 19 | Aminoglycosides | Aminoglycoside N‑acetyltransferases | *AAC6‑PRIME* | 0.1344 | 0.2303 | +0.0959 |
| 20 | Sulfonamides | Sulfonamide-resistant dihydropteroate synthases | *sul1* | 0.0511 | 0.2480 | +0.1969 |

**Supplementary Table S2: Top 20 antibiotic resistance genes (ARGs) detected in the Shitalakshya River water and their temporal dynamics in relative abundance.**The table ranks ARGs by their cumulative relative abundance across both sampling periods (December 2024 and February 2025) and presents the normalized reads per million (RPKM) for each gene. A pronounced shift in the resistome profile was observed between the two time points, with 15 out of 20 ARGs increasing in relative abundance by February 2025. Notably, the macrolide-lincosamide-streptogramin (MLS) resistance gene *MLS23S* decreased substantially, while rifampin (*rpoB*), elfamycin (*tuf*), and aminocoumarin (*parE*) resistance genes showed the most significant increases, coinciding with the overall deterioration in water quality parameters.

**Supplementary Table S3:**

| Gene | Mechanism Group | Expression (December 2024) | Expression (February 2025) | Fold Change | Trend |
| --- | --- | --- | --- | --- | --- |
| *mexK* | RND efflux pump | 0.000 | 0.893 | ∞ | Induced |
| *tetB* | RND efflux pump | 0.418 | 0.881 | 2.11 | Increased |
| *mexD1* | RND efflux pump | 0.311 | 0.639 | 2.05 | Increased |
| *ruvB* | Resistance protein | 0.223 | 0.453 | 2.03 | Increased |
| *qacΔ1* | SMR efflux pump | 0.016 | 0.094 | 5.75 | Increased |
| \|  \| \| --- \|  \| *sitABCD* \| \| --- \| | ABC efflux pump | 0.047 | 0.000 | 0.00 | Decreased |
| *mexT* | RND efflux regulator | 0.105 | 0.000 | 0.00 | Repressed |

**Supplemental Table S3:** **Differential expression of selected multiple compound resistance genes in Shitalakshya River water (December 2024 vs. February 2025).**

**Supplementary Table S4:**

| **Rank** | **Class of Biocide** | **Gene** | **December 2024** | **February 2025** |
| --- | --- | --- | --- | --- |
| 1 | Multi-biocide resistance | *mexW* | 0.0000 | 0.5826 |
| 2 | Peroxide resistance | *sodB* | 0.3260 | 0.5053 |
| 3 | Multi-biocide resistance | *yjcG* | 0.2906 | 0.3223 |
| 4 | Acetate resistance | *lpdT* | 0.2603 | 0.1383 |
| 5 | Multi-biocide resistance | *rpoS* | 0.1896 | 0.1334 |
| 6 | Multi-biocide resistance | *oxyR* | 0.1309 | 0.1316 |
| 7 | Multi-biocide resistance | *tolC* | 0.0623 | 0.0754 |
| 8 | Multi-biocide resistance | *kexD* | 0.0908 | 0.0676 |
| 9 | Phenolic compound resistance | *fabG* | 0.1375 | 0.0896 |
| 10 | Biguanide resistance | *cepA* | 0.0850 | 0.0662 |

**Supplemental Table S4: Top 10 most abundant biocide resistance genes in Shitalakshya River water during December 2024 and February 2025.**
