# Supplementary figures and images for: Seasonal Restructuring of Microbial Communities and Resistomes in the Shitalakshya River, Bangladesh Revealed by Shotgun Metagenomics
Source: Microbiologyopen. 2026 Jul 1;15(4):e70359. doi: 10.1002/mbo3.70359 (PMC13322659; doi:10.1002/mbo3.70359)

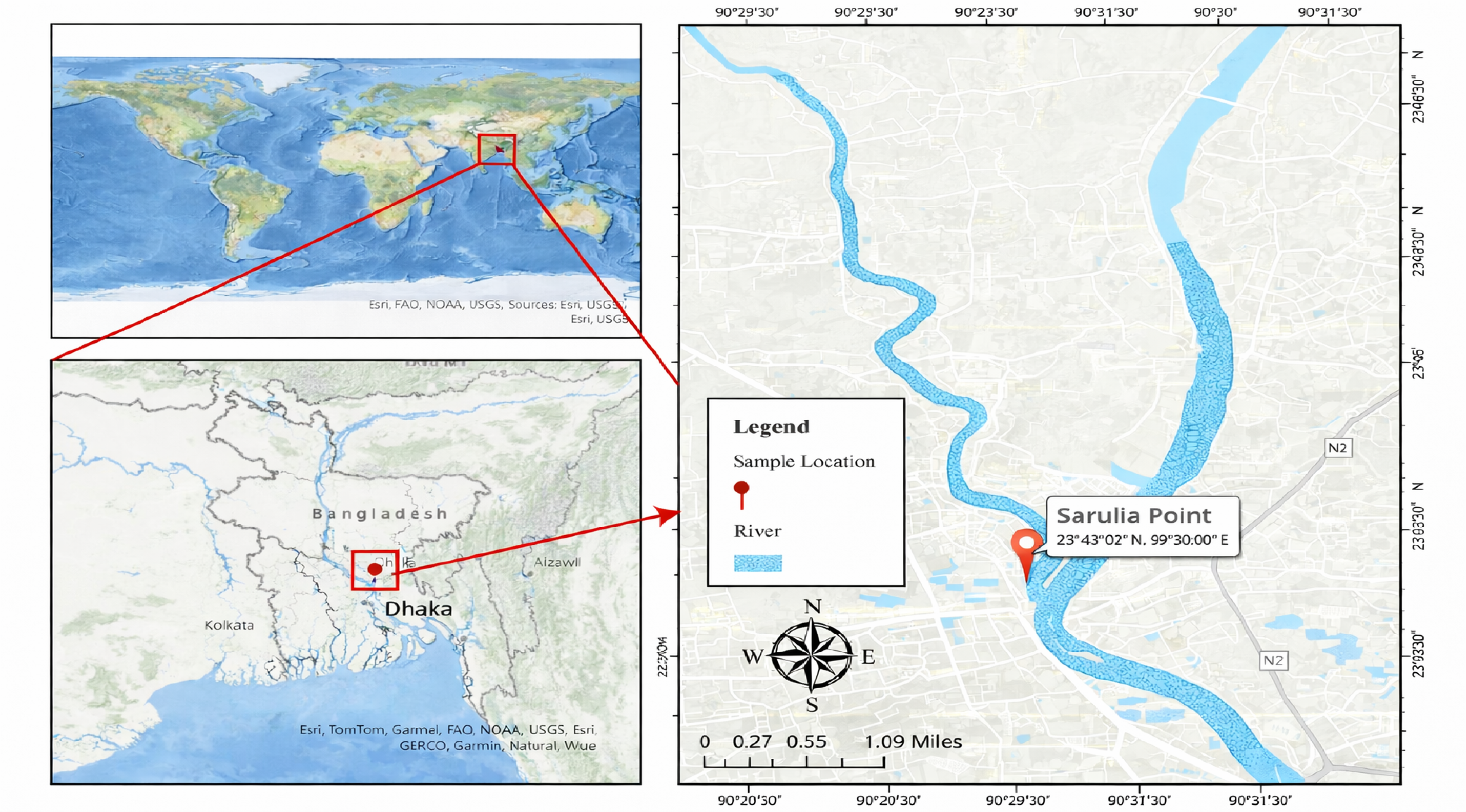

Supplement: Supplementary file 3 — Supporting File 3. [file MBO3-15-e70359-s003.png]
